# Carbohydrate Chemistry II

**DOI:** 10.3390/molecules30183701

**Published:** 2025-09-11

**Authors:** Alberto Marra

**Affiliations:** Institut des Biomolécules Max Mousseron (IBMM), University of Montpellier, 1919 Route de Mende, 34293 Montpellier, CEDEX 5, France; alberto.marra@umontpellier.fr

This Special Issue on carbohydrates, including seven original articles and one review article, is mainly focused on the synthesis of mono, di and oligosaccharide derivatives. In particular, four articles described the preparation [1,2] and activation [3,4] of thioglycosides (*S*-glycosides), a well-known class of carbohydrates which can act as powerful glycosyl donors [5,6,7,8,9,10,11,12] or as chemically and enzymatically stable isosters of the natural *O*-glycosides [13,14,15]. Borbás, Herczeg and their co-workers reported on the multistep syntheses of thioglycosides of four l-hexopyranose, namely l-allose, l-galactose, l-gulose, and l-glucose, from a single very common sugar, the d-mannose [1]. These suitably protected glycosyl donors should provide a straightforward access to the complex, biologically active oligosaccharides and glycoconjugates bearing l-sugar units. A new activation protocol of thioglycoside glycosyl donors was reported by Demchenko and co-workers [3]. Although many efficient promoters were developed in recent years [10,16], these researchers found that CuBr_2_, a commercially available salt less expensive than copper(II) triflate, was able to activate the *O*-benzylated (“armed”) thioglycosides whereas the *O*-acylated (“disarmed”) glycosyl donors also required the presence of 0.5 equiv. of triflic acid. Exploiting their Cu-based protocol, various disaccharides could be prepared in good to excellent yield. Some special thioglycoside glycosyl donors were synthesised by Ando and co-workers and exploited to prepare biologically relevant di and trisaccharides [4]. In fact, they studied the effect of the protecting group at the C-4 position of 3-deoxy-d-manno-2-octulosonic acid (Kdo) α-d-thioglycosides bearing a macrocyclic chain between the carboxylic group (C-1) and the OH-5 function, thus blocking the β-face of the pyranose unit. These glycosyl donors allowed the efficient α-stereoselective glycosylation of primary and secondary sugar alcohols, a chemically and stereochemically challenging reaction due to the lack of anchimeric assistance (neighbouring group participation) and the presence of the strong electron-withdrawing carboxylic ester function [17,18]. Besides the use of thioglycosides as glycosyl donors, the article by Lafite, Daniellou and their co-workers demonstrated the biomedical importance of the *S*-glycosides by enzymatically synthesising a series of coumarin derivatives endowed with interesting fluorescence properties [2]. These new members of the mercapto-coumarin family [19], obtained via trans-glycosidation of *p*-nitrophenyl d-glycopyranosides with 7-mercapto-4-methylcoumarin, were found to be non-cytotoxic and potentially useful as imaging probes.

The present Special Issue also describes some synthetic approaches to carbohydrate derivatives mimicking natural mono, di and oligosaccharides. Aiming to obtain new ligands of a pathogenic *Burkholderia cenocepacia* lectin, Varrot, Bernardi and their co-workers designed, synthesised and tested unnatural, water-soluble anomeric amides of l-fucose and l-galactose that were found to be more active than the methyl *O*-fucoside monosaccharide [20]. These results confirmed that lectins are promising drug targets for anti-adhesion therapy, a powerful tool to prevent bacterial infections or to complement standard antibiotic treatments [21,22,23]. Ishiwata, Takeda and their co-workers prepared a disaccharide constituted of the rare sugars d-allose and d-psicose which mimics the d-sucrose (d-glucose linked to d-fructose), with the aim to replace the latter as a sweetener, thus suppressing the postprandial glucose elevation [24]. The desired α-d-allopyranosidic bond was stereoselectively obtained, taking advantage of the intramolecular aglycon delivery method [25,26,27]. Nifantiev and co-workers synthesised pseudodi, pseudotetra and pseudohexasaccharides structurally related to 1, 2 and 3 repeating units, respectively, of the *Haemophilus influenza* type *a* capsular glycan (*Hia*) [28]. The latter is a linear phosphoglycan featuring d-glucopyranoses β-linked to the d-ribitols. The new pseudosaccharides, obtained by iterative chain elongation using H-phosphonate chemistry [29], were equipped with a spacer which, in one case, was bearing a biotin unit in order to develop a probe for the detection of the *Hia*-associated invasive diseases.

A very important topic, i.e., the glycoconjugate vaccines, was examined in the long review article by Stefanetti and co-workers [30]. This class of vaccines is based on carbohydrate antigens covalently linked to a carrier protein, a feature that allows the sugar antigen to induce a long-lasting immunoglobulin G (IgG) antibody response [31,32,33,34]. The present review examined both the methods to size the polysaccharide antigens and the impact of the glycan chain length on the immunogenicity of the glycoconjugate vaccines based on preclinical and clinical evidence.
